# Meta-analysis of optical low-coherence reflectometry versus partial coherence interferometry biometry

**DOI:** 10.1038/srep43414

**Published:** 2017-02-24

**Authors:** Jinhai Huang, Colm McAlinden, Yingying Huang, Daizong Wen, Giacomo Savini, Ruixue Tu, Qinmei Wang

**Affiliations:** 1 School of Ophthalmology and Optometry, Wenzhou Medical University, Wenzhou, Zhejiang, China; 2 Key Laboratory of Vision Science, Ministry of Health P.R. China, Wenzhou, Zhejiang, China; 3 Morriston Hospital, ABM University Health Board, Swansea, UK; 4 Flinders University, Adelaide, South Australia, Australia; 5 Department of Ophthalmology, No.180 Hospital of Chinese PLA, Quanzhou, Fujian, China; 6 G.B. Bietti Foundation IRCCS, Rome, Italy

## Abstract

A meta-analysis to compare ocular biometry measured by optical low-coherence reflectometry (Lenstar LS900; Haag Streit) and partial coherence interferometry (the IOLMaster optical biometer; Carl Zeiss Meditec). A systematic literature search was conducted for articles published up to August 6th 2015 in the Cochrane Library, PubMed, Medline, Embase, China Knowledge Resource Integrated Database and Wanfang Data. A total of 18 studies involving 1921 eyes were included. There were no statistically significant differences in axial length (mean difference [MD] 0 mm; 95% confidence interval (CI) −0.08 to 0.08 mm; *p* = 0.92), anterior chamber depth (MD 0.02 mm; 95% CI −0.07 to 0.10 mm; *p* = 0.67), flat keratometry (MD −0.05 D; 95% CI −0.16 to 0.06 D; *p* = 0.39), steep keratometry (MD −0.09 D; 95% CI −0.20 to 0.03 D; *p* = 0.13), and mean keratometry (MD −0.15 D; 95% CI −0.30 to 0.00 D; *p* = 0.05). The white to white distance showed a statistically significant difference (MD −0.14 mm; 95% CI −0.25 to −0.02 mm; *p* = 0.02). In conclusion, there was no difference in the comparison of AL, ACD and keratometry readings between the Lenstar and IOLMaster. However the WTW distance indicated a statistically significant difference between the two devices. Apart from the WTW distance, measurements for AL, ACD and keratometry readings may be used interchangeability with both devices.

Accurate ocular biometry is imperative for the management of cataract^1^,^2^,^3^,^4^. The parameters of axial length (AL), anterior chamber depth (ACD), keratometry (K) and white to white (WTW) distance are required in intraocular lens (IOL) power calculation, and for the implantation of phakic IOLs. Inaccurate measurement of AL, ACD, and corneal power contribute to 36%, 42%, and 22%, respectively, of the error in predicted refraction of an IOL using optical biometry^1^,^5^.

The IOLMaster (Carl Zeiss Meditec, Jena, Germany) was introduced in 1999 as the first optical biometer^6^,^7^. This non-contact technology made it possible to evaluate all the required parameters with a single device^8^,^9^. It utilities partial coherence interferometry (PCI) to measure AL with a 780 nm laser diode infrared light. ACD measurement is based on an optical section through the anterior chamber by means of a slit-illumination system with subsequent image assessment. The measurement of K readings in the flat and steep meridian is achieved by analyzing a pattern of light-emitting diodes (LEDs) imaged by the corneal front surface^5^. An image analysis system is used to measure the WTW distance. Each of the IOLMaster’s assessments requires realignment of the device with the visual axis of the eye. This device has been shown to provide highly repeatable measurements regardless of the examiner’s training and accurate measures in both long and short eyes^8^,^9^,^10^,^11^,^12^. In addition, it is also non-invasive and acquisition time is short which has led to the IOLMaster becoming the most popular biometry device used in the clinical setting around the world^13^.

The Lenstar (Haag-Streit, Koeniz, Switzerland), a more recent biometer to the market, has been the first competitor of the IOLMaster. It uses optical low-coherence reflectometry (OLCR) with a 820 nm laser diode infrared light to measure AL and ACD. Similar technology to the IOLMaster is used to measure K and WTW. In comparison to the IOLMaster, the Lenstar is capable of acquiring more parameters, such as corneal thickness and crystalline or intraocular lens thickness^7^,^14^,^15^. Unlike the IOLMaster, the Lenstar is capable of capturing all measurements simultaneously without the need for realignment^14^,^15^.

Many comparative studies have been conducted assessing the differences between common measurement parameters with both these devices. However, there have been some conflicting and inconsistent findings^5^,^9^,^14^,^15^,^16^,^17^,^18^,^19^,^20^,^21^,^22^,^23^,^24^,^25^,^26^,^27^,^28^,^29^,^30^,^31^. This may in part be caused by small study sample sizes, the age or nationality of the patients, use of eyes with or without eye diseases. The aim the present meta-analysis is to combine the results of all comparative studies (IOLMaster versus Lenstar) to determine which parameters are interchangeable.

## Results

### Trial selection

**Figure 1:**
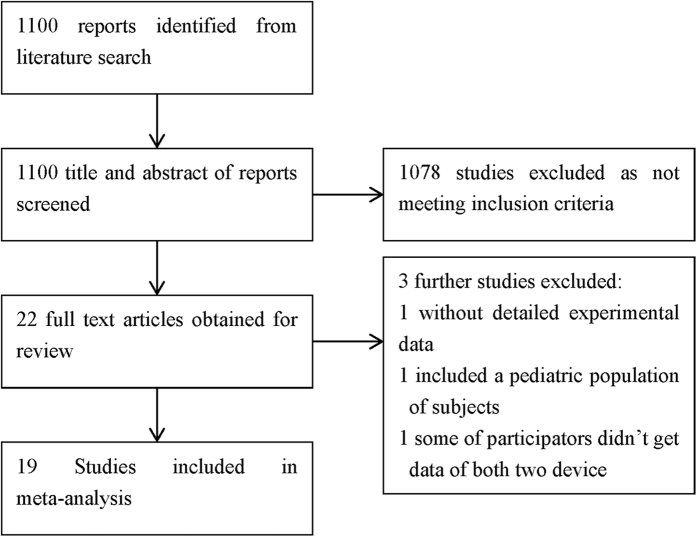
Selection flowchart of the studies included in the present meta-analysis.

The selection flowchart is displayed in Fig. 1. The literature search identified 1100 articles. These 1100 articles and their associated abstracts were screened and 1078 were excluded. The full-text of the remaining 22 articles were obtained and a further 4excluded. The first was excluded due to the lack of the detailed experimental data^32^, second one because patients were children^33^, the third one was some of participators did not obtain data from both devices^26^, and the last one was a duplicate study^30^. Thus, the final 18 trials that met our inclusion criteria were included in the meta-analysis^5^,^9^,^14^,^15^,^16^,^17^,^18^,^19^,^20^,^21^,^22^,^23^,^24^,^25^,^26^,^27^,^28^,^29^,^30^,^31^,^33^.

### Trial characteristics

The characteristics of the eligible studies are summarized in Table 1^5^,^9^,^14^,^15^,^16^,^17^,^18^,^19^,^20^,^21^,^22^,^23^,^24^,^25^,^26^,^27^,^28^,^29^,^30^,^31^,^33^. Some articles lack certain information, such as the proportion of male to female subjects. In such cases, the parameter is marked as unavailable.

### Trial Quality

**Figure 2:**
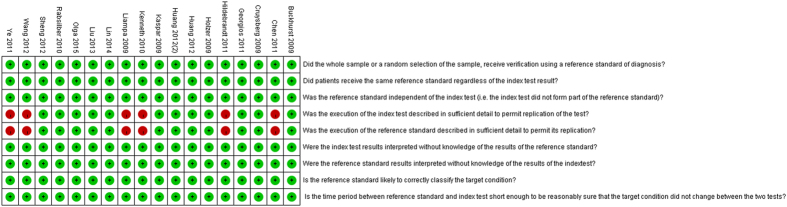
Quality assessment of the included studies.

Quality assessment of the included studies is shown in Fig. 2. Two independent researchers conducted the evaluation. In general, the quality of included studies was high.

### Analysis of AL

**Figure 3:**
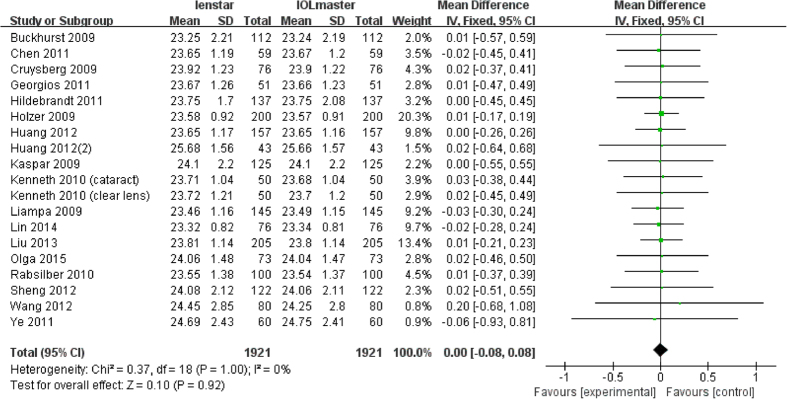
Comparison of AL measurement with the Lenstar and IOLMaster. df = degree of freedom; I2 = extent of inconsistency; Z = overall effect.

All 18 articles measured AL with both devices with a total number of 1921 eyes. The results of the meta-analysis showed that there were no statistically significant differences in the final analysis of AL (MD 0.00 mm; 95% CI −0.08 to 0.08 mm; p = 0.92). The forest plot is shown in Fig. 3.

### Analysis of ACD

**Figure 4:**
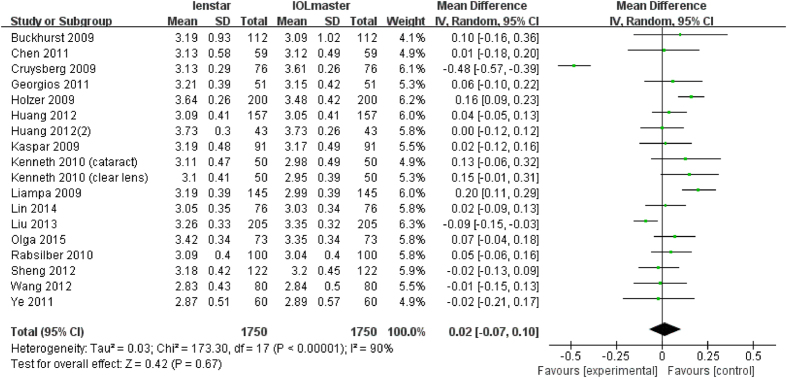
Comparison of ACD measurement with the Lenstar and IOLMaster. df = degree of freedom; I2 = extent of inconsistency; Z = overall effect.

There were 18 articles which measured ACD with a total of 1750 eyes. Liu *et al*. found a statistically significant lower ACD measurement with the Lenstar whereas Holzer *et al*. and Liampa *et al*. found the opposite^16^,^17^,^28^. The meta-analysis showed no statistically significant differences in the final analysis of ACD (MD 0.02 mm; 95% CI −0.07 to 0.10 mm; p = 0.67) between the Lenstar and IOLMaster (Fig. 4).

### Analysis of Kf

**Figure 5:**
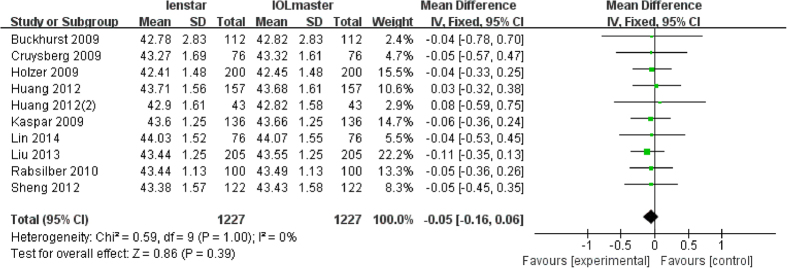
Comparison of Kf measurement with the Lenstar and IOLMaster. df = degree of freedom; I2 = extent of inconsistency; Z = overall effect.

There were 10 articles which measured Kf with a total of 1227 eyes. None of these studies reported a statistically significant difference in the measurement of Kf with both devices. Further, the meta-analysis indicated no statistically significant difference (MD −0.05 D; 95% CI −0.16 to 0.06 D; p = 0.39) (Fig. 5).

### Analysis of Ks

**Figure 6:**
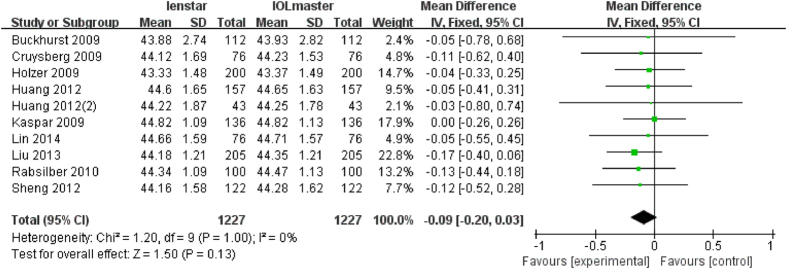
Comparison of Ks measurement with the Lenstar and IOLMaster. df = degree of freedom; I2 = extent of inconsistency; Z = overall effect.

There were 10 articles which measured Ks with a total of 1227 eyes. None of these studies reported a statistically significant difference in the measurement of Ks with both devices. Combining the results, the meta-analysis also showed there was no statistically significant difference (MD −0.09 D; 95% CI −0.20 to 0.03 D; p = 0.13) (Fig. 6), i.e. the Lenstar provides a flatter Ks reading than the IOLMaster.

### Analysis of Km

**Figure 7:**
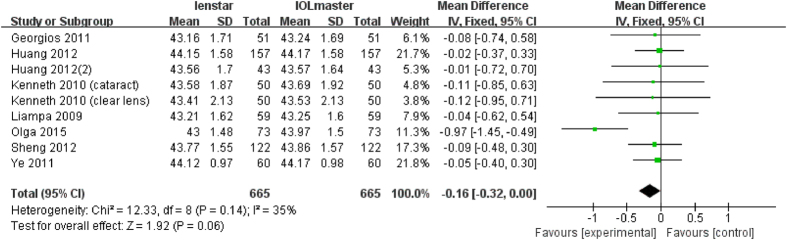
Comparison of Km measurement with the Lenstar and IOLMaster. df = degree of freedom; I2 = extent of inconsistency; Z = overall effect.

There were 10 articles which measured Km with a total of 790 eyes. The study by Olga *et al*. found a statistically significant flatter Km reading with the Lenstar^31^. The remaining 9 studies found no difference. The meta-analysis found no statistically significant difference (MD −0.15 D; 95% CI −0.30 to 0.00 D; p = 0.05) (Fig. 7).

### Analysis of WTW

**Figure 8:**
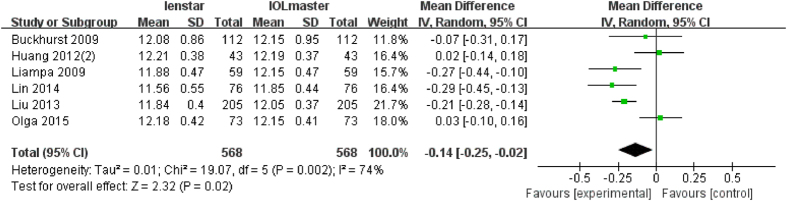
Comparison of WTW measurement with the Lenstar and IOLMaster. df = degree of freedom; I2 = extent of inconsistency; Z = overall effect.

There were 6 articles which measured WTW with a total of 568 eyes. Of these, 3 articles found a statically significant lower WTW distance with the Lenstar^17^,^28^,^29^. The meta-analysis also found a statistically significant lower WTW distance with the Lenstar compared to the IOLMaster (MD −0.14 mm; 95% CI −0.25 to −0.02 mm; p = 0.02) (Fig. 8).

### Sensitivity analysis

**Figure 9:**
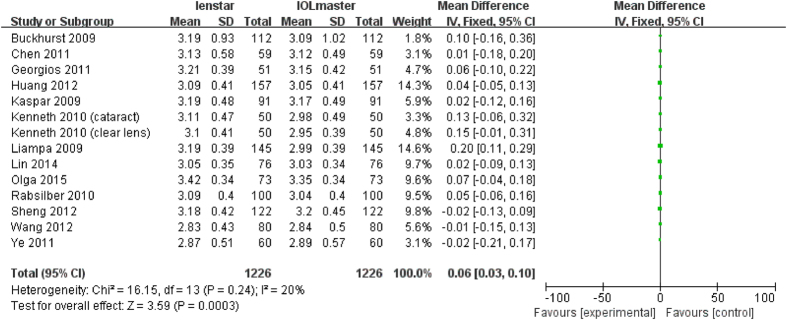
Comparison of ACD measurement with the Lenstar and IOLMaster (mean age > 50). df = degree of freedom; I2 = extent of inconsistency; Z = overall effect.

**Figure 10:**
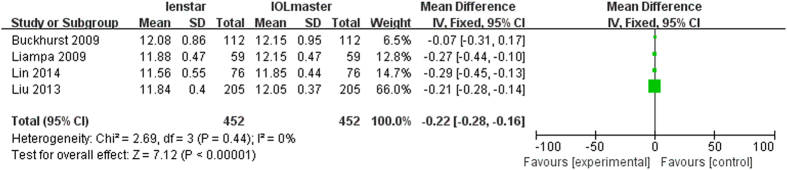
Comparison of WTW measurement with the Lenstar and IOLMaster (exclude Olga2015 and Huang2012(2)). df = degree of freedom; I2 = extent of inconsistency; Z = overall effect.

For each parameter investigated the effect of statistical modeling (fixed-effect model or random-effect model) was evaluated with the results shown in Table 2. The primary heterogeneity of ACD was high, so the sensitivity analysis was performed by excluded Crusberg *et al*.^15^, and the heterogeneity decreased from 90% to 65%, the subjects in the study were found to be younger (all under 50) than in other studies. Another three studies^16^,^23^,^28^ were also excluded since the mean age was younger than 50. The heterogeneity decreased to 20% after those studies (Fig. 9) were excluded. With these exclusions, the overall results did change to become statistically significantly different (p = 0.0003). The same method was performed for WTW. With the removal of two studies: Olga *et al*.^31^ and Huang *et al*.^23^, the heterogeneity decreased from 74% to 0% (Fig. 10). The difference between these two trials and others is that the eyes included had clear lenses whilst the other studies included eyes with lens opacities. The overall results did not change with their removal (p < 0.001).

### Subgroup analysis

Subgroup analyses were performed based on the types of eye diseases. Studies were grouped as either eyes with cataract^9^,^14^,^17^,^19^,^20^,^21^,^22^,^24^,^25^,^26^,^27^,^29^,^30^,^31^,^33^ or other eyes^5^,^15^,^16^,^18^,^23^,^28^. An analysis of the ACD in eyes with cataract indicated a statistically significant (p = 0.001) lower measurement with the IOLMaster. On the contrary, there was a statistically significant (p = 0.006) lower measurement with the Lenstar in other eyes. An analysis of Km indicated a statistically significant (p = 0.04) lower/flatter measurement with the Lenstar in the cataract eyes subgroup (MD −0.16 D) but no statistically significant difference in the other eyes group (p = 0.84). The Lenstar was found to measure a lower WTW distance in the both the cataract and other eyes group (p < 0.0001). Full results of the subgroup analysis is shown in Table 3.

### Publication Bias

**Figure 11:**
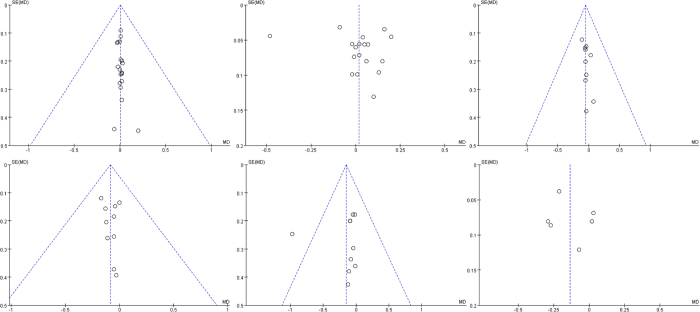
The forest figures of every parameter. The horizontal axis was MD (Mean Difference), ordinates axis was SE (Standard Error).

The publication bias test was performed separately for each parameter. The funnel plot for all parameters is shown in Fig. 11. The funnel plot for the parameters AL, Kf and Ks are symmetry, while other three parameters (ACD, Km and WTW) are not which suggests possible publication bias.

## Dicussion

In the 1990s, ultrasound derivation of AL and manual keratometry measurements were the gold standard in ophthalmology to determine the power of IOL during cataract surgery. The introduction of PCI biometry and automated keratometry measurements was an important step in the field of cataract and refractive surgery^11^,^16^. The IOLMaster was the first device to achieve this in 1999^7^. In more recent years, there have been a number of new non-invasive, non-contact biometers developed, one such is the Lenstar which uses OLCR^34^.

Our meta-analysis evaluates optical biometry measurements between the Lenstar and IOLMaster by comparing the six common measurement parameters. There was no significant difference in AL measurements between the Lenstar and the IOLMaster, and the results indicate excellent agreement between the two devices. Eibschitz-Tsimhoni *et al*. found that a 0.1 mm error value in AL measurement can produce 0.2 to 0.35 D of refractive error^35^. Rabsilber *et al*.^9^, Holzer *et al*.^16^, Hoffer *et al*.^18^ and Buckhurst *et al*.^14^ have all reported a longer AL measurement with the OLCR unit but none of these reached statistical significance. The 95% LoA confirmed the very high level of agreement between the 2 devices^9^,^14^,^16^,^18^. Compared to PCI (laser diode infrared light of wavelength 700 nm) used with the IOLMaster, the OLCR (superluminescent diode infrared light of wavelength 830 nm) used with the Lenstar has a stronger penetrating power and a higher signal-to-noise ratio. In this study, the difference between the two devices machines is small and not of clinical significance.

In this study the results of the meta-analysis indicated that there were no statistically significant differences in the analysis of the ACD measurement. The IOLMaster measures ACD using lateral-slit illumination at approximately 38 degrees to the optical axis. The calculation of ACD is by image analysis of the distance between the corneal epithelium and the anterior surface of the crystalline lens. Further, ACD image analysis as performed with the IOLMaster is also influenced by measurements of the keratometry^5^,^29^. The Lenstar uses OLCR technology superluminescent LED (830 nm) as light. It scans 16 times in one measurement to detect the corneal thickness from epithelium to endothelium and the distance from the endothelium to the anterior surface of the crystalline lens which represents the anatomical ACD^25^. A subgroup analysis of the ACD in eyes with cataract indicated a statistically significant (p = 0.003) lower measurement with the IOLMaster but the opposite was found in eyes without cataract. However the mean difference is of magnitude 0.05 mm which is perhaps not clinically significant. The different results of two subgroups may come from the opacities from eyes with cataract compared to the clear lens in normal eyes.

As for K, the meta-analysis result indicated no statistical significant difference between the two devices. In previous study, Olga *et al*.^31^ found a significant difference in K values (p = 0.031) between the Lenstar and the IOLMaster, but they did not consider the difference to be clinically relevant. The Lenstar takes readings in two circles, 16 points in each circle. The diameter of the inner circle is 1.65 mm, and the outer circle is 2.3 mm. However, the IOLMaster just has one circle which has a diameter of 2.3 mm, and takes readings from 6 points^5^,^29^. Because of more superluminescent EDs imaged on a camera, the Lenstar may have a more repetitive and accurate measurement. In IOL calculation, a 1.0 D measuring error in K-reading may cause an error between 0.9 to 1.4 D in IOL power^22^. A subgroup analysis indicated that the Lenstar measured Km lower/flatter than the IOLMaster in the cataract group. The magnitude of the mean difference was 0.16 D which is perhaps not clinically significant.

The comparison of the WTW distance between the two instruments showed a statistically significant lower measurement with the Lenstar compared to the IOLMaster. Both devices use image analysis systems to measure WTW, but the resolution of the two image analysis systems is different and hence may explain a possible reason for the differences found in this study. The IOLMaster uses slit illumination whereas the Lenstar uses OLCR and a high-definition image sensor. Based on these results, the WTW measurements with the Lenstar and the IOLMaster cannot be used interchangeably. Previous studies have found a similar trend^17^,^28^,^29^.

There are some limitations to this study. The patient groups included those with cataract and normal eyes, although a subanalysis was performed. The performance of these devices in other eye disease such as corneal disease and retinal disease has not been assessed. The study was also limited to adult patients and hence is not applicable to pediatric populations.

In summary, this meta-analysis shows that the Lenstar provides similar results to the gold standard IOLMaster for AL, ACD and K-readings. Hence, both devices can be used interchangeably for these parameters. The WTW distance was found to be different and cannot be used interchangeably.

## Patients and Methods

### Search strategy

A literature search of the Cochrane Library, PubMed, Medline, Embase, China Knowledge Resource Integrated Database and Wanfang Data was performed by J.H.H. and Y.Y.H. The keywords and Medical Subject Headings were “Lenstar” or “IOLMaster” or “optical low-coherence reflectometry” or “optical low-coherence reflectometer” or “partial coherence interferometry” or “partial coherence interferometer”, the time limit was up to August 6th 2015, and the language was not limited.

### Trials Selection

Studies fulfilling the following inclusion criteria were included in the present meta-analysis: (1) Adults; (2) Measurements acquired with both the Lenstar and IOLMaster with the same operator with a short time interval between the two devices; (3) Original data provided in the results section. The full-text of articles with ambiguous titles or abstracts were reviewed for eligibility.

### Parameter Extraction

A customized form for parameter extraction was used to record the study authors, publication year, country of origin, number of patients and eyes, proportion of male to female, average patient age, eye disease and other pertinent parameters to the study recorded.

### Qualitative Assessment

The Quality Assessment of Diagnostic Accuracy Studies (QUADAS) tool was used to assess methodological quality^36^,^37^. The tool is structured as a list of 14 questions which should each be answered “yes”, “no”, or “unclear”. The majority of items included in QUADAS relate to bias (items 3, 4, 5, 6, 7, 10, 11, 12 and 14), with two items each relating to variability (items 1 and 2) and three relating to reporting (items 8, 9 and 13)^36^,^37^. Questions 1, 2, 12, 13 and 14 were assessed to be unsuitable for this study, hence the remaining 9 items were chosen to assess study quality.

### Outcomes

The following common parameters to both the Lenstar and IOLMaster were assessed in this review: AL (mm), ACD (mm), keratometry in the flattest meridian (Kf in diopters [D]), keratometry in the steepest meridian (Ks in D), mean keratometry (Km = (Kf + Ks)/2 in D) and WTW (mm). Original parameters were obtained from the articles as far as possible and parameters that could not be obtained were calculated if possible. Corneal curvatures were analyzed using the refractive index 1.3375 in all but one study which reported a refractive index of 1.332^14^. In this study, the data were recalculated using a refractive index of 1.3375. One article obtained measurements with both devices in a group of 50 subjects with cataract and 50 without cataract^18^. These two groups were assessed separately.

### Statistical Analysis

The data obtained was entered into the RevMan (version 5.2) statistical analysis software. Heterogeneity between studies was assessed by the I^2^ statistic. The 95% confidence interval (CI) estimates were calculated by taking a weighted average of individual study results using a fixed effects model. If the heterogeneity was higher than 50%, the random effects model was used to pool the data. A two-sided *P*-value of <0.05 was considered statistically significant. Subgroup analysis was performed based on eye diseases. Sensitivity analysis was performed by evaluating the effect of statistical model (fixed-effect model or random-effect model) and excluding the potential high heterogeneity studies. A funnel plot and a statistical test for asymmetry was used to assess for the potential for publication bias^38^.

## Additional Information

**How to cite this article**: Huang, J. *et al*. Meta-analysis of optical low-coherence reflectometry versus partial coherence interferometry biometry. *Sci. Rep.* **7**, 43414; doi: 10.1038/srep43414 (2017).

**Publisher's note:** Springer Nature remains neutral with regard to jurisdictional claims in published maps and institutional affiliations.

## Figures and Tables

**Table 1:** Baseline characteristics.

Author and year	Country	Number of subjects	Number of eyes	Male:Female ratio	Mean age (±SD)	Eye disease	Parameters
Olga 2015^31^	Israel	48	73	21:27	63.2 ± 8.75	IOL eyes	AL, ACD, Km, WTW
Lin 2014^29^	China	76	76	35:41	68.1 ± 7.5	Cataract	AL, ACD, Kf, Ks, WTW
Liu 2013^28^	China	205	205	122:83	44.3 ± 5.0	Normal eyes	AL, ACD, Rf, Rs, WTW
Huang 2012^25^	China	98	157	43:55	71.6 ± 7.5	Cataract	AL, ACD, Kf, Ks, Km
Wang 2012^27^	China	40	80	26:14	68.2 ± 7.7	Cataract	AL ACD
Sheng 2012^30^	China	72	122	38:34	64.6 ± 13.4	Cataract	AL, ACD, Kf, Ks, Km
Huang 2012(2)^23^	China	43	43	23:20	22.1 ± 4.7	Normal	AL, ACD, Kf, Ks, Km, WTW
Ye 2011^22^	China	30	60	10:20	62.5 ± 7.7	Cataract	AL, ACD, Km
Hildebrandt 2011^20^	Germany	140	137			IOL eyes	AL
Georgios 2011^21^	Austria	51	51	29:22	68 ± 11	Cataract	AL, ACD, Km
Chen 2011^19^	Austria		59		72.6	Cataract	AL, ACD, Km, WTW
Kenneth 2010^18^	USA	50	50	21:29	74.13 ± 7.12	Cataract	AL, ACD Km
		50	50	24:26	66.22 ± 6.42	Normal	
Rabsilber 2010^9^	Germany	100	100		70 ± 10.6	Cataract	AL, ACD, Rf, Rs
Buckhurst 2009^14^	UK	112	112	36:76	76.4 ± 9.1	Cataract	AL, ACD, Kf, Ks, WTW
Holzer 2009^18^	Germany	100	200		27.25 ± 10.32	Normal	AL, ACD, Kf, Ks
Liampa 2009^17^	USA	145	145		73.5 ± 9.5	Cataract	AL, ACD
Kaspar 2009^5^	America	80	144	34:46	66.9	Mixed	AL, ACD, Rf, Rs
Cruysberg 2009^15^	Netherlands	38	76	25:23	25.9 ± 8.5	Normal	AL, ACD, Rf, Rs

AL = Axial length, ACD = anterior chamber depth, Kf = keratometry along the flattest meridian, Ks = keratometry along the steepest meridian, Km = mean keratometry, Rf = radius of curvature in the flattest meridian, Rs = radius of curvature in the steepest meridian, WTW = white to white, SD = standard deviation.

**Table 2:** Sensitivity analysis performed by evaluating the effect of the statistical model.

Parameters	Fixed-effect model		Random-effect model	
	MD (95% CI)	P	MD (95% CI)	P
AL (mm)	0.00 (−0.08, 0.08)	0.92	0.00 (−0.08, 0.08)	0.92
ACD (mm)	0.00 (−0.02, 0.03)	0.91	0.02 (−0.07, 0.10)	0.67
Kf (D)	−0.05 (−0.16, 0.06)	0.39	−0.05 (−0.16, 0.06)	0.39
Ks (D)	−0.09 (−0.20, 0.03)	0.13	−0.09 (−0.20, 0.03)	0.13
Km (D)	−0.15 (−0.30, 0.00)	0.05	−0.16 (−0.34, 0.03)	0.09
WTW (mm)	−0.16 (−0.21, −0.10)	<0.001	−0.14 (−0.25, −0.02)	0.02

AL = Axial length, ACD = anterior chamber depth, Kf = keratometry in the flattest meridian, Ks = keratometry in the steepest meridian, Km = mean keratometry, WTW = white to white, MD = Mean Difference, CI = confidence interval.

**Table 3:** Subgroup analysis (cataract and other eyes).

Parameters	Overall		Cataract subgroup		Other eyes subgroup	
	MD (95% CI)	P-value	MD (95% CI)	P-value	MD (95% CI)	P-value
AL (mm)	0.00 (−0.08,0.08)	0.92	−0.00 (−0.11,0.11)	0.97	0.01 (−0.11,0.13)	0.85
ACD (mm)	0.02 (−0.07,0.10)	0.67	0.06 (0.02,0.10)	0.001	−0.05 (−0.08,−0.01)	0.006
Kf (D)	−0.05 (−0.16,0.06)	0.39	−0.03 (−0.21,0.15)	0.77	−0.07 (−0.21,0.08)	0.38
Ks (D)	−0.09 (−0.02,0.03)	0.13	−0.09 (−0.27,0.09)	0.32	−0.08 (−0.22,0.06)	0.26
Km (D)	−0.15 (−0.30,0.00)	0.05	−0.16 (−0.32,0.00)	0.04	−0.06 (−0.59, 0.48)	0.84
WTW (mm)	−0.16 (−0.21,−0.10)	<0.001	−0.14 (−0.22,−0.06)	<0.001	−0.17 (−0.24, −0.10)	<0.001

AL = Axial length, ACD = anterior chamber depth, Kf = keratometry in the flattest meridian, Ks = keratometry in the steepest meridian, Km = mean keratometry, WTW = white to white, MD = Mean Difference, CI = confidence interval.
